# Depredation of domestic herds by pumas based on farmer’s information in Southern Brazil

**DOI:** 10.1186/1746-4269-10-73

**Published:** 2014-10-15

**Authors:** Francine Schulz, Rodrigo C Printes, Larissa R Oliveira

**Affiliations:** Laboratório de Ecologia de Mamíferos, Universidade do Vale do Rio dos Sinos (UNISINOS), Av. Unisinos, 950, São Leopoldo, RS 93.022-000 Brazil; Laboratório de Gestão Ambiental e Negociação de Conflitos, Universidade Estadual do Rio Grande do Sul (UERGS), Rua Assis Brasil, 842, Sao Francisco de Paula, RS 95400-000 Brazil; Grupo de Estudos de Mamíferos Aquáticos do Rio Grande do Sul (GEMARS), Rua Machado de Assis, 1456, Osório, RS 95520-000 Brazil

**Keywords:** Protected Areas, Human-wildlife conflict, Predation on domestic herds, *Puma concolor*, Southern Brazil

## Abstract

**Background:**

Large carnivores such as pumas are frequently killed due to conflicts with human populations involving predation on domestic herds. In Southern Brazil, traditional pasture systems, where animals feed without specific husbandry practices is typical, becoming the herds vulnerable to puma attacks. The aim of this study was to examine the conflict between local people and pumas in a Protected Areas mosaic in southern Brazil.

**Methods:**

Forty-five face-to-face interviews with local people were performed during the year of 2011, using a structured questionnaire with open and closed questions about puma attack episodes in some farms. Based on responses, the conflict and puma attacks were described, and the characteristics of attacked farms and estimated financial losses were evaluated. The first respondents were indicated by the Local Environmental Agency, and the others were indicated by the first one and so on, which is known as “snow-ball” method.

**Results:**

Our data suggested that pumas used to attack in unfavorable conditions of visibility (foggy days) and on easier prey (e.g. sheep). Most of the attacks reported were close to forested areas and were focused on free herds during feeding activities. Some farmers said they gave up their sheep breeding activity due to losses caused by puma attacks. However, some farmers could over estimate their losses. Moreover, pumas were considered a threat to domestic herds and respondents mentioned cases of illegal puma hunting in the area. The results of questionnaires suggested that puma attack episodes were related to fragmentation of their habitat associated to incorrect management of herds in the farms studied. The diagnosis of this type of conflict and the characterization of most attacked sites are extremely important to create strategies to prevent and control attacks by wild carnivores.

**Conclusions:**

Deep changes in husbandry practices added to educational programs should be implemented, in order to maintain the sustainability of rural activities as well as the survival of pumas in southern Brazil.

**Electronic supplementary material:**

The online version of this article (doi:10.1186/1746-4269-10-73) contains supplementary material, which is available to authorized users.

## Background

Cultures across the world have over time developed characteristic ways of interacting with the environment and regional flora and fauna [1]. Ethnobiology can use popular knowledge and attitudes as an instrument for the conservation of natural resources [2]. One of the ethnobiological tools are interviews guided by structured questionnaires that seek to translate the respondent’s thoughts and actions toward the researched object [3]. Ethnozoology, as a branch of this science, studies the roots as deep within the past as the first relationships between humans and other animals, evaluating the variety of interactions that human cultures maintain with animals [4]. Even hunting activity can be studied in order to analyze conflicts between people and animals [5–10]. In recent years, the importance of ethnobiological studies for biodiversity conservation has increasingly been recognized [1].

Despite the relevance to conservation biology of conflict between people and wild animals, this theme has been little explored in an ethnobiologic perspective. Even with a known strong interaction between Brazilian fauna and local populations, ethnobiological studies are scarce. A recent review analyzed the distribution of publications (scientific papers, books and book chapters) from 1939 to 2011 [4]. A total of 487 studies were published up to July 2011 and Zootherapy – the use of animals and their sub-products in folk medicine – was the majority of the studies (17.86%). Education and management represented 3.7% of the papers and only 2.87% were on Ethnomastozoology. In relation to biomes analyzed in this study, Atlantic Forest had 5.75% papers and southern region of Brazil only 2.0%, indicating the absence of ethnobiological information in the area [4].

Carnivores such as large cats are extremely important to maintain the ecologic equilibrium, once they guarantee the diversity and resiliency of ecosystems. As predators, they can help controlling herbivore populations (top-down effect on trophic cascades) [11] and as they usually leave behind a great part of their prey for several motives, they provide food to the maintenance and diversity of scavenger and decomposer communities [12]. Large carnivores are killed worldwide especially due to conflicts involving these animals and rural communities [13–15]. Moreover, felids have always been subject of human fascination and fear, generating a huge historic of conflicts [16, 17]. Species like lions (*Panthera leo*), tigers (*Panthera tigris*), jaguars (*Panthera onca*) and pumas (*Puma concolor*) have lost their natural habitats and preys, which lead them to coexist with human populations and domestic herds [18]. As domestic animals have lost most of their natural instincts, they are easy prey for wildcats [19]. This has become a serious conservation problem for these species, which have been severely threatened. Studies agree that the contact of rural populations with large felids has resulted in purposeful elimination of these animals that are preventively killed due to depredation of domestic herds or as a hunter’s trophy [20–22]. This kind of conflict is one of the most urgent wildcat conservation issues worldwide and the human perceptions and interactions with these animals need to be studied to the conservation of felids populations [17].

A review of several studies showed that the high mortality rate of adult pumas (75.00%) is due to conflicts with humans [23]. Puma depredations on livestock are being studied in countries such as USA (e.g. [24]), Canada (e.g. [25]), Mexico (e.g. [26]), Venezuela (e.g. [27]), Bolivia (e.g. [28]), Argentina (e.g. [29]) and Chile (e.g. [30]).

In Brazil, pumas coexist with jaguars and both are being hunted by farmers in retaliation to depredation of domestic herds. Jaguars usually kill animals larger than pumas such as eight-ten month-old calves. Pumas mostly kill sheep, goats and younger calves [19]. They usually kill their prey biting the back of the neck (smaller prey), and eventually suffocating it biting their throats (larger prey) [31]. Feeding starts soon after the ribs, which can even be broken. The stomach and intestines are usually removed without disruption. Liver, lungs and heart are commonly eaten. The muscles of the hind legs are usually the next part to be consumed. Partially consumed carcasses are commonly covered with organic material as dried leaves, for protection against other animals and for future feeding [13].

Some studies on this type of conflict are being conducted in different regions of Brazil such as Pantanal [32], Amazonian Forest [15, 33], Cerrado [34] and Atlantic Forest [20, 35]. In Southern Brazil, conflicts between humans and pumas have been observed in the *Foz do Iguaçú* National Park [36] and in the coastal mountains of southern Brazil, between states of Santa Catarina and Rio Grande do Sul [21, 37].

The Southern region of Brazil has a very agricultural tradition. The state of Rio Grande do Sul, where this study was carried out, is one of the most degraded states of Brazil, with just few remaining forest areas. In this state, the population of pumas has been reduced to only a few individuals, inhabiting mainly the most escarped edges of the North-eastern plateau and the region at the border with Argentine forests [16, 38].

In the attempt to solve ecological problems and to protect and restore its biological diversity, Brazil’s government created in 2000 the National System of Protected Areas (Federal Law 9985/00). The creation of most Brazilians Protected Areas was problematic and caused conflicts with local communities, especially because people who live within the areas have to change attitudes and habits in order to protect the environment [39].

In this sense, this study describes the conflict between local people and the depredation by pumas on livestock in the southern region of Brazil and quantify the economic losses caused by these felines in the Atlantic Forest. Based on information given by respondents throughout interviews guided by a standard questionnaire, suggestions for future conservation plans in Protected Areas in the region could be made.

### The local setting

#### Study area

**Figure 1:**
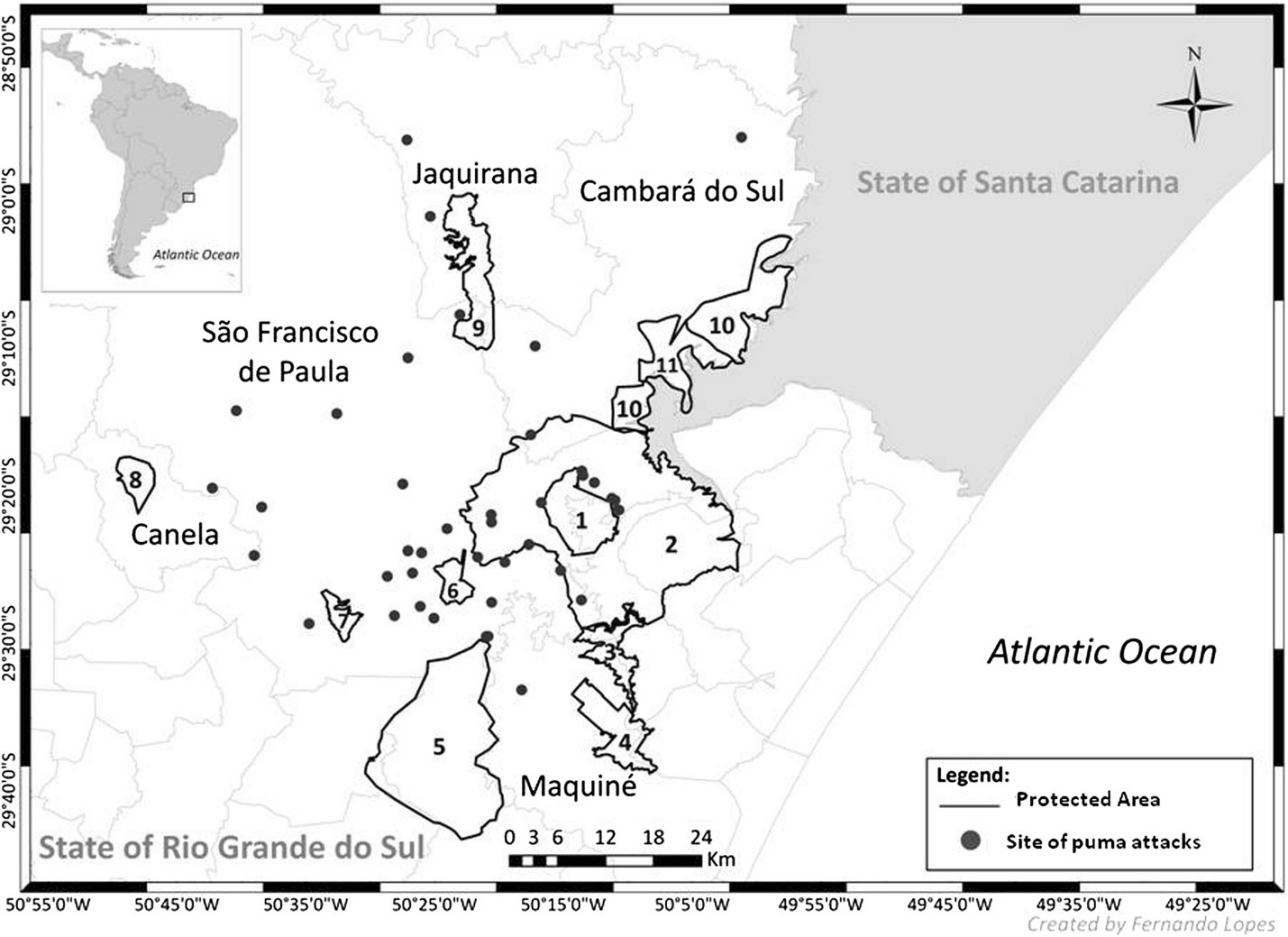
**Site of puma attacks, where each number refers to a different Protected Area: 1 –** ***Aratinga*** **State Ecological Station; 2 –** ***Rota do Sol*** **State Environmental Protected Area; 3 –** ***Pró-Mata*** **Centre for Research and Nature Conservation; 4 –** ***Serra Geral*** **State Biological Reserve; 5 –** ***Riozinho*** **State Environmental Protection Area; 6 –** ***São Francisco de Paula*** **National Forest; 7 –** ***Ronda*** **Municipal Park; 8 –** ***Canela*** **National Forest; 9 –** ***Tainhas*** **State Park; 10 –** ***Serra Geral*** **National Park; 11 –** ***Aparados da Serra*** **National Park.**

The study was conducted in a Protected Areas mosaic in North-eastern state of Rio Grande do Sul, Brazil, approximately from 50°01’W to 50° 42’W and from 28°55’S to 29°33’S (Figure 1). In this region, there are at least 11 Protected Areas, including two National Parks and two National Forests. These areas belong to the Atlantic Forest Biosphere Reserve, which represents 17.00% of the state of Rio Grande do Sul. It was created in order to preserve natural resources and to avoid the increasing deforestation of the Atlantic Forest [40].

The area belongs to the Atlantic Forest Domain, which is characterized by plateaus surrounded by steep cliffs, with slopes at the eastern part covered by the Atlantic Forest up to 700 m and Araucaria Forest at 700–1600 m. As a consequence of anthropogenic fire used to expand the livestock area in the region, the fields above the mountain divide the landscape with forest remnants. The annual average temperature is between 14°C and 20°C and annual precipitation rates are relatively high, more than 2.000 mm. At the highest part of the region, near the upland top, fog is a constant phenomenon [40].

The regional economy is mainly based on forestry, traditional livestock and small agricultural practices. Extensive system is applied to livestock practices that demand low economic investments and natural factors are determinant in the productive processes. The demographic density is low with large areas of pastures and plantations. The education level is also low. However, currently the younger residents are going to school and becoming a channel of communication between rural communities and the city [40].

The local population has as source of income livestock and logging of pines, generating for approximately 200 years, which is considered a very traditional lifestyle based on rural practices. Currently we can notice this kind of traditions in the local cuisine and in the traditional parties and rodeos [40]. The cities located in the study area have about U$ 151.7 millions of GDP in which the services sector accounted for 52.80% of GDP, followed by agricultural sector (24.70%) and industry sector (19.00%). We can see the importance of the agricultural sector comparing to the numbers of the Rio Grande do Sul state, where the agricultural sector represents 12.30% of the GDP. The total population of the study region represents 0.405% of the population of all state and the local GPD represents only 0.313% of the total state GPD [40].

## Methods

### Data collection

The study was approved by an Ethical Committee (protocol number: 051/2011), according to a Brazilian Federal Law (CNS 196/96) that treats studies that involves humans’ population.

We conducted 45 face-to-face interviews with local farmers in the year of 2011 guided by a standard questionnaire (see Additional file 1) with open questions (several words to be answered) and with closed semi-structured questions (which can be answered with few words or with yes/no answers) [3, 41]. As a criteria, we put our questions in a sequence in order to make respondents more comfortable and confident to give honest answers about delicate questions [3, 33], such as the killing of pumas in the region. This question was placed by the end of the questionnaire. At the end of interviews, an illustrative board with four pictures of felines – jaguar, puma, ocelot (*Leopardus pardalis*) and jaguarondi (*Puma yagouaroundi*), was shown to the respondents to test if they could correctly identify a puma. All this strategy was used to check if they really knew the animal that they were talking about. Other studies used the same methodology [15]. It is important to mention that the jaguar is extinct in the region for a long time. Therefore, there is no cultural confusion about the species jaguar and puma in the midst of the local population.

All the answers given by respondents were related to the years of 2008 until 2011.

The language used in the questionnaire was similar to that of respondents. The interviews were conducted as a dialogue in order to create interaction and trust between interviewer and respondent, avoiding doubts about the answers. The notes taken were as faithful as possible, keeping the language and information given by respondents. Shirts with conservationist slogans and with drawings of animals were not dressed for the interview to avoid false or induced answers.

As a starting point to the study, three Protected Areas were selected to the first contacts: the *Rota do Sol* State Environmental Protection Area (29°23’22.58”S; 50°11’3.90”W), the *Aratinga* State Ecological Station (29°18’S and 29°24’S; 50°11’W and 50°17’W) and the *Tainhas* State Park (29°05’35.33”S; 50°21’51.55’W). The managers of these Protected Areas were contacted and indicated the first persons to be interviewed. This methodology is known as “snow ball” [42] and is based on the respondents’ indication by the first interviews - which are previously designated by a reference group. This methodology is very useful to select the informers and investigate one problem in a fragment of a larger geographic area [43, 44].

The frequency of puma attacks was characterized to the respondents as the number of times that pumas attacked herds in their farms within one year. The distance from the site where pumas usually attacked herds was divided in five categories: less than 50 m, between 50 and 100 m, between 101 and 500 m, between 501 and 1.000 m and more than 1.000 m, considering five different sites: house, native forest, paved road, dirt road and pine plantation.

Forest edge was previously defined to the respondents as the region just a few meters away from the forest.

After the interviews, an informed consent form was given to each respondent to be signed in two copies: one copy was kept with the interviewed and the other with the respondent. This form explicitly reported that the respondent provided all information freely and that everything that has been said is kept in secrecy and would be used only for research purposes.

Following the methodology [21], the Rural Union, the Rural Association and the Technical Institute of Rural Enterprise of the state of Rio Grande do Sul (*EMATER -* in Portuguese) were contacted to evaluate the average price of the local herds, in order to calculate the financial losses of respondents who had their herds attacked by pumas in their farms between 2008 and 2011. For this calculation, the number of animals killed (according to information provided by respondents) was multiplied by the reference value obtained by the average (calf = U$ 255.68; sheep = U$132.39; goat = U$132.39; young horse = U$ 549.43 and swine = U$48.30). The dollar exchange rate was calculated using the average rate (U$ 1 = R$ 1.76) in the year when the interviews were conducted in Brazil (2011).

The distance between the georeferenced property and the first closest Protected Area was given by the Google Earth program, tracing a straight line from the property to the boundary of the closest Protected Area.

### Data analyses

All data collected were quantitatively analyzed. Quantitative approach was used to measure some aspects such as the geographic characteristics of farms and sites where pumas attacked, respondents’ economic and social profile, financial losses, sort and number of herds, climate characteristics when the attacks occurred and type of management applied to herds in the farms. The frequencies of answers from open and closed questions were calculated to each question category, according to the following formula: Frequency = number of times that one answer was given x 100 / total number of answers. It is important to explain that for open questions, the most repeated answers were analyzed by their frequency.

Some hypotheses were also tested using the Person’s Correlation, Kruskal-Wallis and Qui-Square tests to verify if: 1) there was correlation between the distance from the nearest Protected Area and the number of dogs in the property, total area of properties, total forest area in the property, altitude, herd size, puma attack frequency and number of heads predated; 2) there was correlation between number of farmers who gave up breeding any kind of herd and puma attack frequency; 3) there was a correlation between seasons, climate conditions, type of herd and puma attack frequency in the properties and 4) there was association between puma attack frequency and the distance from the site where the attacks occurred to native forest and type of management used with sheep flocks.

## Results

Forty-five interviews were personally conducted in the study area, but only 42 geographic coordinates were collected (Figure 1). The interviews were mostly conducted in the municipality of São Francisco de Paula especially due to the presence of rural establishments (Rural Union and Rural Association). Twenty-six interviews were conducted at the farms affected by puma attacks. Twenty-nine interviews were carried out with the owners of the establishments, 13 with their wives or sons and three with herd keepers.

### Respondents’ profile

Thirty-nine respondents were male and six were female. The respondents aged between 41 and 80 years and most of them had 30 years of residence in the area.

The predominant educational level was high school followed by incomplete basic education, graduation and complete basic education.

The predominant occupation and main source of income was based on agricultural practices, mainly cattle’s breeding. The average monthly income was considerably low for fifteen respondents who declared to earn between one and two regional minimum wage (U$ 312.50 and U$ 625.00). Eight respondents earned between U$ 937.50 and U$1,250.00; 12 respondents earned between U$ 1,562.50 and U$1,875.00; and ten respondents earned more than U$ 1,875.00 dollars monthly. The medium monthly income per person in the state is U$ 647.43 [45].

### Adjacent area and properties’ characteristics

Among all the properties with coordinates taken, 27 were inside at least one Protected Area Buffer Zone, 12 of them belonged to *Rota do Sol* State Environmental Protected Area or to *Aratinga* State Ecological Station. Only three properties were not part of a Buffer Zone or near the perimeter of some local Protected Area.

The distance between the nearest Protected Area and the property searched was not correlated with the puma attack frequency inside the properties (r = 0.21; p > 0.358) nor with the number of animals killed (r = 0.17; p = 0.291).

The altitude was homogeneous and the average altitude was about 883 m. Altitude was not correlated with the puma attack frequency (r = 0.11; p = 0.159) neither with the number of animals killed (r = −0.12; p = 0.113).

The largest property searched had 1.310 hm^2^ and the smallest had 6.00 hm^2^, and the average size of all properties searched was 400.40 hm^2^ (SD = 386 hm^2^). The average size of native forest within the farms was 104 hm^2^. The largest area of native forest in a property was 500 hm^2^ and the smallest was 0.50 hm^2^ (SD = 136 hm^2^).

All respondents said they have natural water sources to the herds. This indicates that breeding in an extensive system with free access to native forest is intimately associated with natural water sources.

The majority of respondents had more than one dog in their properties. The average number of dogs in each property was four, but they did not live with the herds. In some properties dogs where next to the place where the herds used to spend the nights. The breeds were: mongrel (112), collie (30), German shepherd (15), Australian shepherd (03) and others (15). However, the number of dogs per property and the puma attack frequency did not show linear correlation (r = −0.19; p = 0.373), and was not correlated with the number of animals killed (r = −0.09; p = 0.061).

Most respondents had more than one type of herd, and cattle’s breeding was associated with other smaller herds. The herds in most of the properties had not a specific breed and usually were for meat (n = 26), followed by milk (n = 5) or both (n = 13). Only two of the 45 farmers did not have cattle. All type of herds were breed free in the field, with access to the forest to protection, feeding and watering. Just small herds of sheep, goats, horses and pigs were protected in closed areas during the night. According to the farmers, they started to do that after the first pumas attacks in their properties. Some farmers also used to bring the cow with their new born near to the houses to avoid the attacks. From 45 farmers, 26 had sheep herds and only one did not have cattle’s breeding in association. Nineteen sheep breeders had herds with less than 50 animals and seven had herds ranging from 50 to 199 animals. Among all sheep breeders, thirteen adopted extensive management and thirteen adopted semi-extensive management of their herds. Thus, 13 properties did not have any nocturnal protection. Therefore, the type of management showed no association with the puma attack frequency (*X*^2^ = 1.75; g.l. = 2; p = 0.417).

The total number of animals, considering all types of herd, showed no linear correlation with the puma attack frequency (r = 0.04; p = 0.080). However, a positive correlation was found between the number of domestic animals killed in the properties (from 2008 to 2011) and the total number of animals (r = 0.39; p = 0.010). In other words, farmers with larger herds had more losses.

There was a dependence on the type of herd attacked by pumas and the frequency of these attacks (Kruskal-Wallis, Dunn method, H = 16,66; p = 0.002), where sheep were more predated than goats and pigs.

### Losses due to puma attacks

Forty-three of all respondents had episodes of depredation by pumas in their properties. Fifteen respondents had losses in more than one year searched and twenty-eight had animals killed by pumas only in one specific year.

Taking into account all attacks reported by respondents, the following scenario of predation by pumas in the study area was obtained: 26 properties attacked in 2008, 18 in 2009, 16 in 2010 and 12 in 2011.

**Figure 2:**
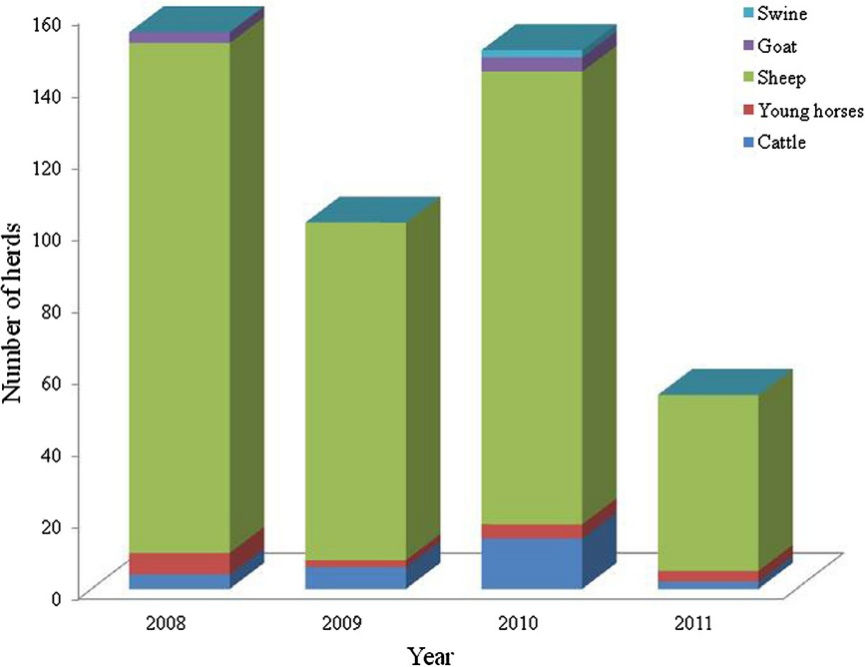
**Number and type of herd predated by pumas per year.**

The total number of animals killed in all properties was 155 animals in 2008, 102 animals in 2009, 150 animals in 2010 and 54 animals in 2011 (Figure 2). Three respondents did not know how many animals were lost by attacks in 2008 and two respondents did not know their losses in the year of 2009. Thus, the total loss caused by pumas based on local prices was U$ 23,515.34 in 2008, U$ 15,077.27 in 2009, U$ 22,915.91 in 2010 and U$ 8,646.59 in 2011. According to respondents, the total loss for the entire period corresponded to U$ 70,155.11 (considering all types of herds). The largest loss was reported by sheep breeders, who had more cases of puma attacks (Table 1). We did not count the losses due to abortion, animal mass reduction and stress that can be provoked by puma attacks episodes.

**Table 1:** **Type of herds and reported losses during the years 2008 to 2011**

Year	Type of herd	Losses (U$)	Total (U$)
2008	Cattle	1,023	23,515
	Young Horses	4,620	
	Goat	397	
	Sheep	17,475	
	Swine	0	
2009	Cattle	1,534	15,077
	Young Horses	1,099	
	Goat	0	
	Sheep	12,444	
	Swine	0	
2010	Cattle	3,580	22,917
	Young Horses	2,198	
	Goat	265	
	Sheep	16,681	
	Swine	193	
2011	Cattle	511	8,646
	Young Horses	1,648	
	Goat	0	
	Sheep	6,487	
	Swine	0	
TOTAL (U$)			70,155

Twenty-five respondents who had losses due to puma attacks gave up breeding sheep and goats because, according to them, it is not profitable to change husbandry practices to avoid puma attacks. Most of respondents said that because of puma attacks people are stopping to breed sheep in the region, leaving their ancient culture and changing it to cattle’s breeding. The farmers sustain that cattle requires less attention and precautions in general. There was a positive correlation between the number of farmers who gave up the breeding activity and puma attack frequency (r = 0.98; g.l. = 2; p = 0.024). However, the question about the main causes of herd’s mortality in general had 75 answers where diseases were the most cited (n = 32) followed by carnivores attacks (n = 16).

All respondents declared never had killed a puma, but 25 said they heard about pumas’ killing in the area. Twenty-four reported the hunting of wild animals such as birds and small mammals inside and near their properties.

### How people identified puma attacks

Among the 43 respondents who reported puma attacks in their properties, only two were not sure if a puma was really the animal responsible for the depredation. One thought it could be a boar and the other one did not find the carcass to blame a specific predator.

On the other hand, 41 respondents guaranteed that a puma was the responsible for their losses. When questioned about how they knew about the author of the attacks, there were 56 different answers, including the feline’s characteristic footprints (the most cited) and the way how the carcasses were disposed and consumed by the predator. According to respondents, pumas do not leave nail marks on the ground and usually eat the neck region, rips, lungs, liver, heart and the bottom of carcasses. Only ten respondents visualized the puma attacking or consuming the hunted prey.

Forty respondents found the carcasses of killed animals. According to 26 of them, carcasses were not covered with leaves and sticks and according to 14 respondents, carcasses were covered with this type of material.

When questioned about what they did with the carcasses, 33 respondents answered they left it where they found it, seven buried the carcasses and two cooked them because they were still fresh.

Ninety answers were given by respondents about how the carcasses were consumed. The neck region (n = 24) and internal organs like heart, liver and lungs (n = 23) were the most cited.

At the end of the interview when the illustrative board was shown, 42 of 45 respondents could identify the puma according to the ecological literature about this animal. One person pointed to a jaguar and two persons pointed to a jaguarondi.

### Characteristics of puma attacks

**Figure 3:**
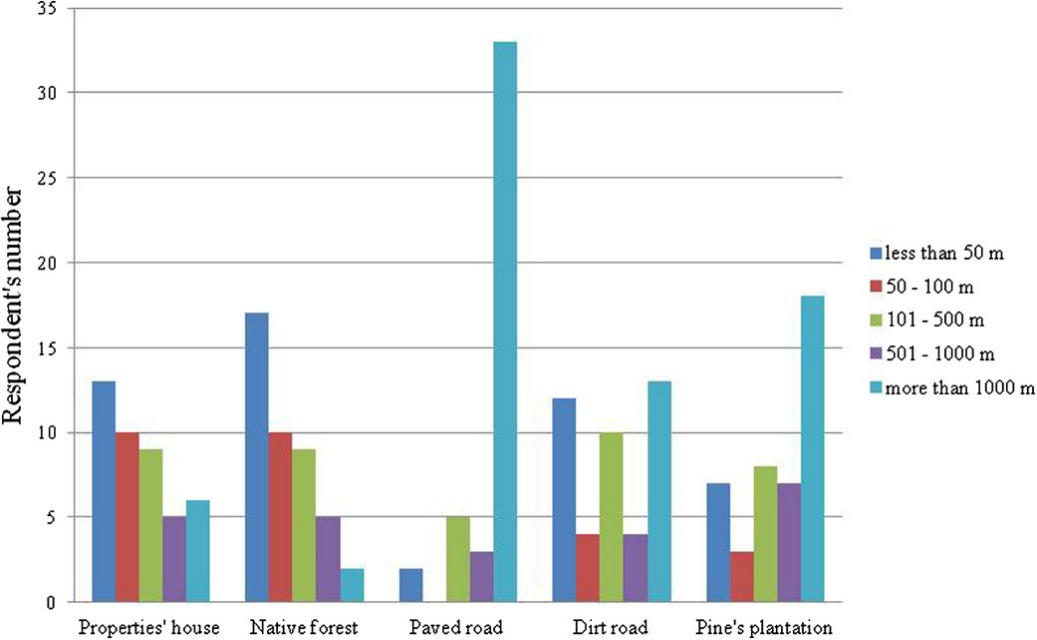
**Distance informed by respondents of sites where puma attacks occurred.**

Most respondents declared that the distance of sites where pumas used to attack is more than 1.000 m from de first paved road and less than 50 m from the nearest native forest (Figure 3).

There was no association between the distance of the nearest native forest from the site where pumas used to attack domestic herds and the puma attack frequency, considering all the five categories described in methodology (*X*^2^ = 3.30; g.l = 8; p = 0.914), nor considering all attacks within 100 m from the native forest as a distinct category and compared with all attacks far more than 100 m from the native forest as another distinct category (*X*^2^ = 0.70; g.l = 2; p = 0.704).

The typical vegetation cited by respondents as the site of puma attack corresponded to 44 answers, being defined as forest edge by 21 respondents.

Respondents reported that attacks occurred mostly at night, mentioned 35 times. The time that the attacks occurred accounted for 49 answers: dawn was reported by 20 respondents, twilight by 14 respondents, afternoon by three respondents and morning by two respondents. Ten respondents did not remember the time when the attacks occurred.

The question about the season that most attacks occurred in the same property corresponded to 69 answers; winter was cited 20 times, summer 19 times, spring 15 times and fall 12 times. Only three respondents did not remember the season that the attacks occurred. However, there was no dependence between season and the puma attack frequency (H = 2.09; g.l. = 3; p = 0,553).

The weather conditions in the days when the attacks occurred had 100 answers, considering years from 2008 to 2011; fog was cited 52 times, sun was cited 29 times and rain was cited 18 times. Only two of the 43 respondents did not remember how the weather was in the days when pumas attacked. There was correlation between weather conditions and the puma attack frequency: foggy days had more puma attack episodes when compared with sunny or rainy days (H = 7.47; g.l. = 2; p = 0.024).

## Discussion

The interviews showed that local people and pumas have a coexistence conflict in the study area, due to puma attacks to domestic herds. We believe that the information collected throughout structured questionnaires, even being an indirect way and not our own personal observation on pumas attacks, is a very precious data about species and the conflict with local farmers. Based on the information given by a group of people, it is possible to get sufficient evidences to create a general scenario of the reality in a specific area [44].

Several studies suggested that people can exaggerate in the number of domestic animals killed by wild predators. It has been historical cited in different regions of the world for pumas [46], wolves (*Canis lupus*) [47, 48], lynx (*Lynx lynx*) [48], bears (*Ursus arctos*) [48], snow leopards (*Panthera uncial*) [47], jaguars [22] and some many others predators. In this sense, we are aware that maybe some farmers could overreact, resulting in an overestimation of their herd losses due to the puma attacks. However, it is impossible to precisely calculate this effect on our data. In order to avoid bias on this data we asked them how to recognize a puma attack (based on carcasses marks) and how to describe a puma track. Most of the respondents answered correctly mentioning the typical signals: big tracks without nail marks close to the animal killed, bites on neck and rips region, besides the consumption of internal organs such as lungs, liver and heart [13, 31].

Moreover, most of the interviewed farmers discarded the possibility of foxes (*Cerdocyon thous* and *Pseudalopex gymnocercus*) and wild (and domestic) dogs attack on their herds based on different marks on the carcasses. These answers were consistent with literature about pumas and canids feeding patterns, suggesting that most of the farmers had enough knowledge about these predators’ habits. In this context, we believe that interview methods as metric instrument to document conflicts with predators in rural areas are very efficient to map the general scenario of conflicts in any region of the world. The communities that coexist with large carnivores need to be heard and understood in order to create real management strategies. Other studies found the same kind of conflicts between carnivores, reptiles and birds where these species are hated and hunted due to the conflicts with human’s populations [1, 4–10, 13–15].

Several studies have shown that the losses caused by wild carnivores are low when compared to other general causes of mortality, reaching maximum rate of 3.0% [15, 20, 32, 34]. In our study respondents even cited that diseases in general were the main cause of their herd’s mortality. However, even representing low loss, attacks by carnivores need to be monitored and minimized to preserve these species and to avoid illegal killing in Protected Areas, near and outside them, searching to restore and maintain viable populations of these animals [22, 27].

The data obtained did not allow finding a correlation between puma attack frequency and the total area of forests and altitude of farms. One possible explanation to this fact is the influence of surrounding landscapes, which can work as a green corridor to the movement of pumas in the region. Nevertheless some authors [21, 37] found that higher altitude, vegetation and slope are factors that can increase the number of puma attack episodes.

There was no correlation between the distance from the first Protected Area and puma attack frequency. However, the geographic coordinates showed that most attacks occurred in a farm within a Protected Area or in a buffer zone. This homogeneity probably affected the statistical analysis.

The number of dogs in the properties was not correlated with puma attack frequency either, but most dogs were not with or near the herds when the attacks occurred. Guard dogs are efficient only when they are trained and kept with the herds, chasing carnivores [49].

The losses caused by puma attacks determined the decision to stop breeding sheeps. Sheep were more affected by depredation episodes, being the herd most attacked by pumas from 2008 to 2011. If the respondents did not give up the sheep breeding activity, they reduced the number of flocks, because according to them, it would not be profitable building a sheepfold to keep the sheeps safe from the attacks of pumas, especially when there are a large number of animals. Pumas were probably responsible for a decrease of about 70.00% in the sheep breeding activity in the state of Santa Catarina, near the study area, especially due to the extensive management applied in the area [50].

Considering that most respondents gave up or reduced their herds since the attacks of pumas started to increase (2008), a decrease in losses and in the puma attack frequency in the properties was observed. Based on respondents’ reports, it could be inferred that the higher frequency of puma attack to sheep flocks brought changes in management practices, which possibly mitigated losses caused by them. Reducing the herd allowed respondents to keep their sheep near their properties and sometimes in a closed place at night, when attacks by pumas are more frequent. This kind of practice seems to reduce the frequency of attacks and losses caused by pumas. It is important to mention that changing the husbandry practices from extensive to semi-extensive management did not reduce conflicts with farmers, only reduced the depredation by pumas.

Possibly, this change from extensive management to semi-extensive management, after puma attacks, influenced the result of statistics due to the lack of association between type of management and puma attack frequency. Therefore, those who answered to have a semi-extensive management probably had an extensive management when most attacks occurred in the property. As the attacks were accounted all together for the statistical analyses (from 2008 to 2011), the properties that changed their husbandry practices were categorized according to the type of management used during the year when the interviews were conducted (2011).

It is noteworthy that cattle’s breeding was reported as the main activity in the properties and the largest losses were related to sheep, thus, when pumas start to attack cattle’s calves, the conflict with local population could become more serious. A six-month-old calf is worth approximately double of an adult sheep and the cattle’s breeding management is extensive and without a controlled breeding season in the region. This problem needs more attention from environmental authorities.

According to the farmers interviewed, the site where pumas used to attack was near the properties’ house and near native forest areas. According to another Brazilian study, pumas have shown better toleration to the presence of humans than jaguars, for example, moving across crowded areas in order to search for prey [15]. Moreover, the areas that overlap human populations and pumas has increased contact and conflicts between them, due to the predation on domestic herds by pumas [22].

According to respondents, the availability of water source to the herds was always related to some natural resource near forest areas where the herds have free access, becoming more vulnerable to the attack by carnivores such as pumas. Forest edges were also reported as typical site of puma attacks. It seemed that domestic animals that are more vulnerable were more attacked in the farms studied. Several studies have demonstrated that the proximity of forest areas and the inadequate management of herds, allowing the entrance of domestic animals inside the forests to feed and to drink water increase depredation by pumas and other carnivores [15, 21, 34, 51].

On the other hand, puma attacks occurred far from paved roads. Possibly, these animals try to avoid open areas that do not offer adequate camouflage and protection like forest areas do [52].

Most attacks by pumas occurred at night, in the dawn and in foggy days. This fact can suggest that pumas prefer conditions of low visibility, avoiding human contact. In addition, pumas seem to chase their prey in condition of vulnerability, spending less energy in the hunt and avoiding fractures caused by prey. Predators in general try to balance the benefits and losses from their hunts, searching an optimal foraging, minimizing time and energy to feed [19].

The puma attack frequency was not dependent on a particular season, but related to weather conditions. The constant fog formed in the region, regardless of season, can explain it. The pumas in the state of Santa Catarina also used to attack in similar conditions, avoiding contact with humans and hunting in favorable conditions when prey are more vulnerable [21].

The fact that respondents left the carcasses where they were found can stimulate pumas to return to the place to feed again later, having also the opportunity to attack the herds again. Some manuals to avoid and minimize attacks by carnivores suggest among other things to remove and to bury the animals killed [19, 53].

A diagnosis of this type of conflict and the characterization of favorites sites where attacks occur are extremely important to create strategies to prevent and control attacks by wild carnivores [1, 4, 15, 17]. In the context of the conflict caused by puma attacks in the study region, mechanisms to reduce the economic losses and educational programs about this feline should be proposed by environmental and agricultural organs. In this sense, the results and suggestions on the management of herds at the properties and the characteristics of puma attacks presented in this study should be considered for future strategies in the region, in order to maintain the sustainability of rural activities as well as the survival of pumas in southern Brazil.

## Acknowledgements

We would like to thank SEMA-RS for the use of local facilities to conduct the field research, Silvio Marchini for reviewing our questionnaire, Fernando Lopes who gently did our Figure 1 and Fabrícia Barbieri for the scientific support during the work. We are also grateful to Cleusa Schulz, Éverson Fleck, Fernanda Zardo, Gabrieli Schulz, Jaqueline Ramos, Marcela Zini, Mathias Melo and Roque Santos for collaborating in the field work. Finally, we would like to thank all respondents who shared with us their time and information.

## Electronic supplementary material

Additional file 1: **Standard Questionnarie.** (DOC 470 KB)
