# Supplementary material for: Depredation of domestic herds by pumas based on farmer’s information in Southern Brazil
Source: J Ethnobiol Ethnomed. 2014 Oct 15;10:73. doi: 10.1186/1746-4269-10-73 (PMC4271476; doi:10.1186/1746-4269-10-73)
Supplement: Supplementary file 1 — Additional file 1: Standard Questionnarie. (DOC 470 KB) [file 13002_2014_443_MOESM1_ESM.doc]

**STANDARD QUESTIONNARIE**

1. GENERAL INFORMATION

1.1. Protocol number: _______________

1.2. Date: _____/_____/_____

1.3. City: ___________________________________________________________________

1.4. Nearest town: ____________________________________________________________

1.5. Distance: ________________________________________________________________

1.6. Population: ______________________________________________________________

1.8. Is the property inside a Protected Area? ( ) no ( ) yes

1.9. Wich one? ______________________________________________________________

1.10. Is the property in the Buffer Zone of a Protected Area? ( ) no ( ) yes

1.11. UTM COORDINATE (Datum SAD 69):______________________________________

1.12. Property altitude (m): _____________________________________________________

1.13. Owner’s name: __________________________________________________________

1.14. Telephone number: ______________________________________________________

2. PROFILE OF INTERVIEWED:

2.1. Fisrt name and nickname: __________________________________________________

2.1. Gender: _________________________________________________________________

2.3. Age: ___________________________________________________________________

2.4. Lifetime in the region: _____________________________________________________

2.5. Education Level: _________________________________________________________

2.6. Occupation: _____________________________________________________________

2.7. Monthly income (media): ___________________________________________________

3. ADJACENT AREA AND PROPERTIES’ CHARACTERISTICS:

3.1. Pasture Area (ha):_______ Cultivation Area (ha):________ Forest Area (ha):_______ Total Area (ha):______________

3.2. Type of cultivations: ( ) forestry ( ) potato ( ) winter pastures ( ) other

3.3. What kind of? ____________________________________________________________

3.4 Property’s main activity: ____________________________________________________

3.5. Native Forest? ( ) no ( ) yes

3.6. Type of source of watter avaiable to herds: ( ) spring ( ) river ( ) weir ( ) other option What kind of? _______________________________________________________________

3.7. Are there dogs in the property? ( ) no ( ) yes How many?

3.8. Breeds:_________________________________________________________________

3.9. What kind of mammals (or their traces) can be found inside the property?

___________________________________________________________________________

3.10. Is there hunting activies inside the property? ( ) no ( ) yes

3.11. Most common hunted animals: _____________________________________________

4. HERDS’ CARACTERISTICS:

4.1.Type of herds in the property:

| Cattle  Number: | Horses  Number: | Goat  Number: | Sheep  Number: | Swine  Number: |
| --- | --- | --- | --- | --- |

4.2. Type of management: ( ) extensive (free herds) ( ) semi-extensive ( ) intensive (closed herds)

4.3 Where the herds used to spend the nights? ______________________________________

4.4. Main procdution system: ( ) meat ( ) milk ( ) calving

4.5. Avarege mortality of herds during one year: _______cattle ________horses

_______goats ________sheeps ________swines

4.6. Main Causes of these mortalities: ____________________________________________

______________________________________________________________________________________________________________________________________________________

5. CHARACTERISTICS OF PUMAS ATTACKS:

5.1. Did you have any loss in your herds because of a wild animal attack from the year of 2008? ( ) no ( ) yes

5.2. Do you know which wild animal was? ( ) no ( ) yes

5.3. Which was the animal? ( ) puma ( ) ocelot ( ) wild cat ( ) other _______________

5.4. Did you see the wild animal attacking? ( ) no ( ) yes

5.5. How many times did you see the animal attacking? ______________________________

5.6. How do you know which was the animal? _____________________________________

5.7. What is the colour of a puma? _______________________________________________

5.8. What is his average size (meters)? ____________________________________________

5.9. How many times a puma attacked the property in each year? 2008______2009______2010______2011_______

5.10. How many domestic herds were attacked in each year? 2008______2009______2010______2011_______

5.11. Caracteristics of the place where the attcks occured:

5.11.1. Distance until the property’s house (m): _____________________________________

5.11.2. Distance until the nearest Native Forest (m): _________________________________

5.11.3 Distance until the nearest paved Road (m): ___________________________________

5.11.4. Distance until the nearest dirt Road (m): ____________________________________

5.11.5. Distance until the nearest Pine’s plantation (m): ______________________________

5.12. Vegetation cover in the area where the attacks occured (most of the times): ( ) native forest ( ) pasture/forest (in the forest bordery) ( ) dirt pasture ( ) clean pasture ( ) other Wich one? ________________________________________________________

5.13. Day-shift (most frequent): ( ) day ( ) night hour: __________________________

5.14. Most frequent season when the attacks occurred: ___________________________________________________________________________

5.15. Most frequent climate conditions when the attacks occured: ( ) rainy days ( ) sunny days ( ) foggy days ( ) there were not preferences

5.16. Did you see the carcass of the animals killed? ( ) no ( ) yes

5.17. Number of times that you sow the carcasses: __________________________________

5.18. How the carcasses were covered? By leaves or sticks? ( ) no ( ) yes

5.19. Number of carcasses covered: _________ 5.18. Number of carcasses uncovered: ______

5.20. What did you do with the carcasses (most of times)? ___________________________________________________________________________

5.21. Which carcasses parts were eaten (most of the time)? ___________________________________________________________________________

5. 22. What did you do after the attacks occured?

___________________________________________________________________________

## 5.23. Did you stop to breed some kind of herd because of pumas attacks?

## ( ) no ( ) yes

5.24. Have you ever heard about pumas killing in the region?

( ) no ( ) yes

5.25. Have you ever tried to kill a puma?

( ) no ( ) yes

#### 6. ILUSTRATIVE BOARD

6.1. Which of these animals is the puma? A ( ) B ( ) C ( ) D ( ) Didn’t recognise ( )

6.2. Have you ever seen one of these animals? ( ) no ( ) yes

6.3. Wich one? A ( ) B ( ) C ( ) D ( )

6.4. Do you know their names?

A ________________________________________________________________________________

B ________________________________________________________________________________

C ________________________________________________________________________________

D ________________________________________________________________________________

##### PRANCHA ILUSTRATIVA


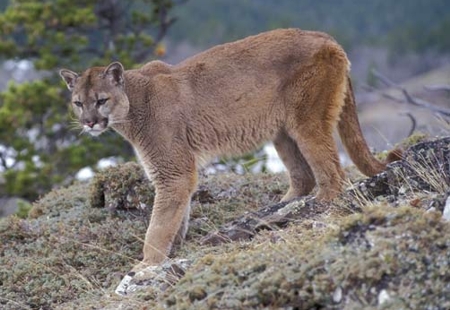

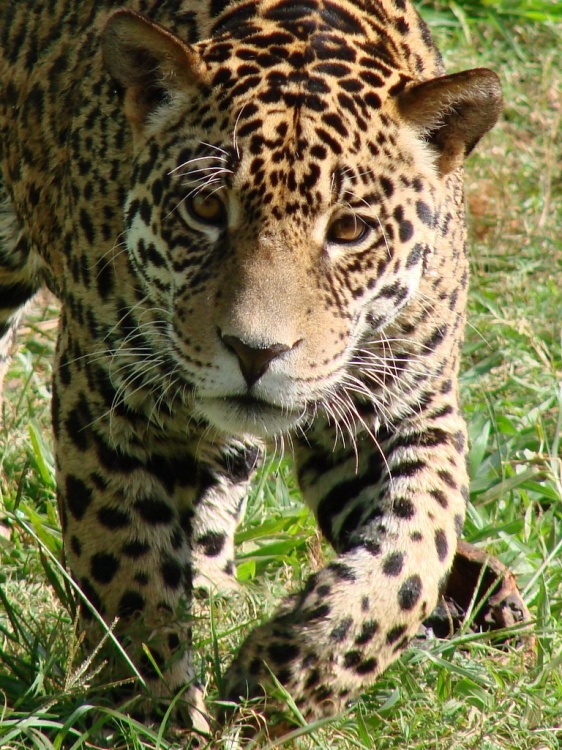


A

B


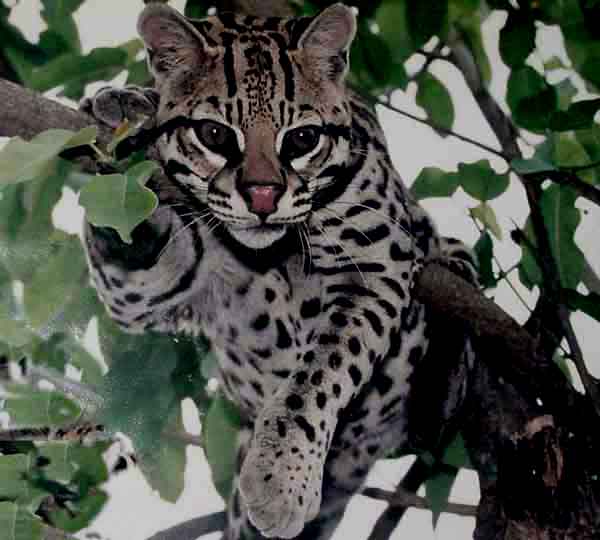

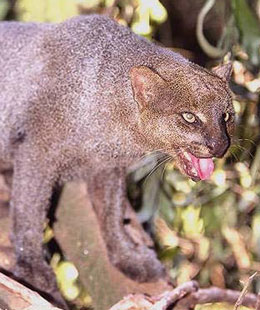


C

D

C
